# Chronic Manganese Neuro-Toxicity in a Patient With Cirrhosis and the Role of Iron-Deficiency Anaemia

**DOI:** 10.7759/cureus.75198

**Published:** 2024-12-06

**Authors:** Jamal Jamal, Muhammad Hashmat, Hasan Al Alwan, Eleanor Harvey, Mansoor Zafar, Panagiotis Stamoulos, Gayatri Chakrabarty

**Affiliations:** 1 Gastroenterology, East Surrey Hospital, Surrey and Sussex Healthcare NHS Trust, Redhill, GBR; 2 Internal Medicine, East Surrey Hospital, Surrey and Sussex Healthcare NHS Trust, Redhill, GBR; 3 Radiology, East Surrey Hospital, Surrey and Sussex Healthcare NHS Trust, Redhill, GBR; 4 Gastroenterology, Surrey and Sussex Healthcare NHS Trust, Redhill, GBR; 5 Gastroenterology and Hepatology, Surrey and Sussex Healthcare NHS Trust, Redhill, GBR

**Keywords:** atypical parkinsonism, cirrhosis, manganese neurotoxicity, metabolic dysfunction-associated steatotic liver disease (masld), non-alcoholic fatty liver disease (nafld)

## Abstract

Patients with chronic liver disease (CLD) are prone to complications associated with impaired liver functioning. This coupled with iron-deficiency anaemia (IDA) can predispose them to multiple comorbidities. We present an interesting case of a 69-year-old woman with a background history of liver cirrhosis due to metabolic dysfunction-associated steatotic liver disease (MASLD) with frequent travels to Bangladesh, a southeast Asian country known for having high levels of manganese (Mn) in water for domestic use. She presented with progressively worsening cognition and extrapyramidal symptoms. She underwent routine blood tests including for liver functions followed by a non-contrast computed tomogram (CT) of the head that did not suggest a possible cause. However, magnetic resonance imaging (MRI) of the brain showed hyperintensities bilaterally in the globus pallidus, subthalamic nucleus, red nucleus, and substantia nigra and raised the possibility of Mn toxicity. This was confirmed with repeated raised blood levels of Mn. The query was raised for acute toxicity to Mn, followed by consideration of CLD history associated with reduced elimination of Mn. This was complicated further by her history of IDA. Her case was discussed in a multi-disciplinary setting with specialities including radiology, gastroenterology, neurology, psychiatry, hepatology, and elderly medicine. Following this, a decision was made for the best supportive management of the patient. This case highlights the importance of MRI in the detection of a rare case of Mn toxicity, in a predisposed individual contributing to cognitive decline with extrapyramidal symptoms.

## Introduction

Manganese (Mn) is a trace element mineral that human bodies cannot produce. A small amount of Mn in food or supplements is necessary as a cofactor for various enzymes including pyruvate carboxylase, manganese superoxide dismutase and arginase [1,2]. This cofactor function enables successful carbohydrate, cholesterol, and amino acid metabolism. Additional roles encompass reproduction, hemostasis, immunity, elimination of reactive oxygen free radicals, and osteogenesis [3,4]. Mn has been reported to be absorbed throughout the small intestine, predominantly ileum, although some studies have suggested increased absorption via the duodenum [1,2]. Following absorption of Mn, it may remain free, or bound to alpha 2 macroglobulin, transferrin, or albumin. The total body content of Mn is 10- 20 mg in organs such as the liver, brain, kidney, and pancreas while about 20-45 per cent (%) remains in the bones [1,2]. The normal whole-body Mn level is usually maintained at 4 to 15 mcg/L [1] with mean serum concentrations ranging from 0.6 to 4.3 micrograms per litre (μg/L) in healthy adults [5-7]. The absorption and excretion are regulated [6] with the excretion of the great majority of absorbed Mn in faeces via bile (>90%) and a small amount excreted via urine [1,4].

Foods rich in Mn include mussels, with 5.8 grams/serving (per cent daily value of 252), potatoes, with 0.3 grams/serving (per cent daily value of 13), and brewed coffee, with 0.1 grams/serving (% daily value of 4) [8]. Mn toxicity has been reported in patients exposed to inhalation of high amounts of Mn dust from mining and welding professions and people consuming water with higher Mn concentrations of as high as 28 milligrams per litre (mg/L) [9]. People with chronic liver disease (CLD) have limited ability to excrete Mn in bile and can be at risk of toxicity of Mn [6]. Iron-deficiency anaemia (IDA) also increases Mn absorption and may exacerbate symptoms of Mn toxicity [2].

Mn toxicity can affect the central nervous system, and common symptoms include unsteady gait, hearing loss, tinnitus, rigidity, and tremors [1,2]. The psychosomatic symptoms include an exhaustive list of insomnia, anorexia, irritability, depression, mania, and delusions. Often the symptoms may seem to be extrapyramidal, mimicking Parkinsonian syndromes [1,6,9].

Groundwater remains the single largest source of drinking water in most developing countries of Southeast Asia, including Bangladesh [10,11]. In such countries, iron (Fe), Mn, and Lead (Pb) play significant roles in water pollution [12].

According to the joint national hydrochemical survey by the British Geological Survey and the Department of Public Health Education in 2001, about 42% of tube wells have Mn concentrations exceeding permissible limits. Unlike the eastern part of Bangladesh, the groundwater of central, northern, and western Bangladesh seems to be highly Mn-contaminated [13].

We describe a 69-year-old female patient with a background history of liver cirrhosis from metabolic dysfunction-associated steatotic liver disease (MASLD) formerly non-alcoholic fatty liver disease who presented with neurotoxicity due to high exposure to Mn. Initially, there was suspicion of acute Mn intoxication. However, collateral history suggested a more chronic picture of perhaps reduced levels of Mn clearance on a background of CLD. This may have stemmed from repeated exposure to drinking water with high levels of Mn during a previous history of frequent trips over the years to a remote residence in northeastern Bangladesh. This, coupled with liver cirrhosis, may have led to Mn neurotoxicity.

## Case presentation

A 69-year-old woman was admitted to a district general hospital for progressive confusion, weakness, and fatigue. Discussion with the family suggested a gradual decline in her cognition and functional status since an episode of reactive psychosis four years ago.

She had a past medical history of diabetes mellitus type 2, reactive depressive psychosis, hypertension, reflux oesophagitis, MASLD with cirrhosis, Fibro scan liver stiffness measurement (LSM) 75 kilopascal (kPa), controlled attenuation parameter score of 400 decibels per meter (dB/m), with portal hypertension with grade 1 oesophageal varix, gastric antral vascular ectasia (GAVE) and associated IDA. 

Her measured weight was 82.95 (kilograms) kg, her height was 149.3 (centimetres) cm and her body mass index (BMI) was 37.21 kilograms per meter square (kg/m^2^). Her medications included empagliflozin tablet oral 10 mg once a day (OD), ferrous sulfate oral 200 mg three times a day, Gaviscon Advance oral suspension liquid oral 5 mL to 10 mL as required for indigestion, lansoprazole capsule (enteric-coated) oral 30 mg OD, lurasidone tablet oral 37 mg OD, mebeverine oral 135 mg three times a day, vortioxetine tablet oral 10 mg OD, and spironolactone tablet oral 25 mg OD.

**Figure 1:**
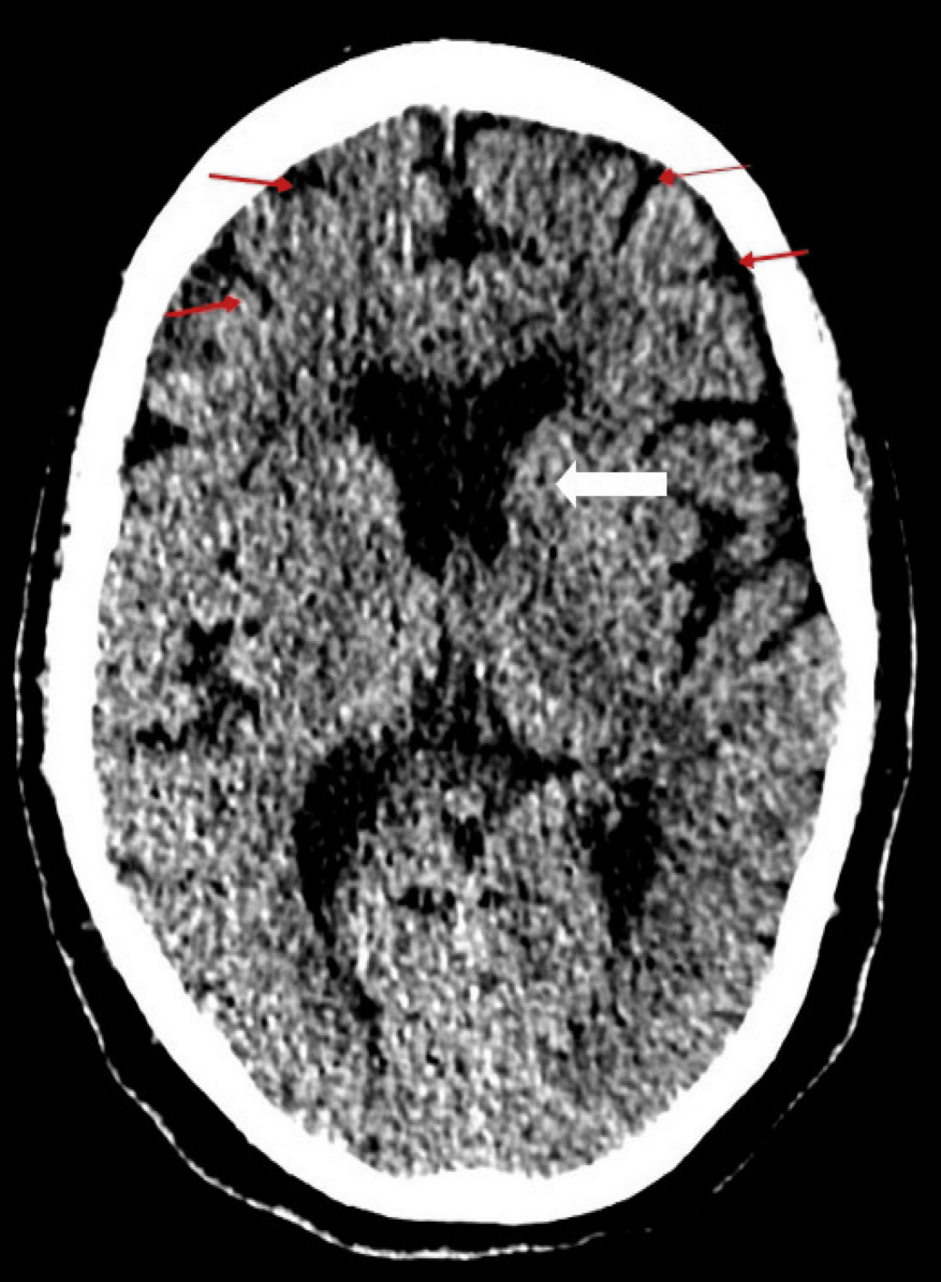
Axial computed tomogram (CT) head demonstrating a subtle old infarct in the left caudate nucleus (white arrow) and generalised brain involution signifying brain atrophy (red arrows).

On examination, the Glasgow coma scale (GCS) score was 14/15 (eye response 4/4, motor 6/6, verbal response 4/5) due to mild confusion. Neurological examination revealed a symmetric akinetic-rigid form of spasticity with shuffling gait and no resting tremors. Further assessment suggested she may have hepatic encephalopathy with a West Haven Grading score of 2/4 and a Rockwood Dalhousie University Frailty scale score of 5/9. She had a computed tomogram (CT) of her head that showed only mild chronic cerebral microvascular disease including an old infarct in the left caudate nucleus and, a few small old infarcts in the left caudate nucleus with mild generalised brain atrophy (Figure 1).

She underwent frailty and memory blood tests including ammonia levels, vitamin B12, folate, vitamin D, thyroid-stimulating hormone (TSH) levels, and human immune deficiency virus (HIV) screening. The blood tests with routine serum electrolytes, urea, creatinine, and liver function tests (LFTs) along with her modified end-stage liver disease (MELD) and Child-Pugh score estimation are shown in Table 1.

**Table 1:** Blood test results for the patient showed iron deficiency anaemia, reduced serum albumin, and a raised serum ammonia level with a modified end-stage liver disease (MELD) score of 6 and a Child-Pugh score of 8B. ^*^eGFR, estimated glomerular filtration rate; ^** ^CRP, C- reactive protein; ^!^ TSH, thyroid stimulating hormone; ^!!^ T4, thyroxine; ^!!!^ T3, triiodothyronine; ^$^Vitamin B12, Cyanocobalamin; ^$$^ HIV, human immunodeficiency virus; ^&^25 OH Vitamin D, Cholecalciferol

Parameter	Units of measurement	Reference	Patient’s results
Sodium (Na)	mmol/L	133-146	137
Potassium (K)	mmol/L	3.5-5.3	4.1
Urea	mmol/L	2.5-7.8	4.1
Creatinine	mmol/L	49-90	66
eGFR *	ml/minute/1.73 m^2^	90-120	82
Albumin	(g/L)	35-50	22
Alkaline phosphatase (ALP)	IU/L	30-130	85
Alanine transaminase (ALT)	IU/L	0-55	44
Aspartate transaminase (AST)	IU/L	0-34	70
Bilirubin	mmol / L	0-20	14
Ammonia	mmol / L	0-45	105
CRP ^**^	(mg/L)	0-5	1.5
Iron (Fe)	mmol/L	9-30	5
Transferrin	g/L	1.8-3.2	3.2
Transferrin saturation	(%)	15-50	6
Ferritin	mg/L	30-250	8
Adjusted calcium	mmol/L	2.2-2.6	2.38
TSH ^!^	mIU/L	0.35-4.94	2.32
T4 ^!!^	pmol/L	9-19.1	9.2
T3 ^!!!^	pmol/L	2.4-6	3.3
Folate	mg/L	3-20.5	14.8
Vitamin B12 ^$^	ng/L	200-900	1070
HIV ^$$^ 1 & 2 Antibody and p24 Antigen		-	negative
25 OH Vitamin D ^&^	nmol/L	50-200	59
Haemoglobin A1c (HbA1c)	mmol/mol	20-41	33
Haemoglobin (Hb)	g/L	115-165	99
Mean cell volume (MCV)	fL	75-105	94.5
Red cell count	(10^12^/L)	3.5-5.5	3.23
Mean cell haemoglobin concentration (MCHC)	g/L	290-350	324
Red cell distribution width (RCDW)	%	11-15	30.7
Erythrocyte sedimentation rate (ESR)	-	0-40	109
Platelets	10^9^/L	150-450	202
White cell count	10^9^/L	4-11	4.3
International Normalisation Ratio (INR)	-	1	1
Modified end-stage liver disease (MELD) score	-	6-40	6
Child-Pugh score	-	5-15	8 (Child class B)

**Figure 2:**
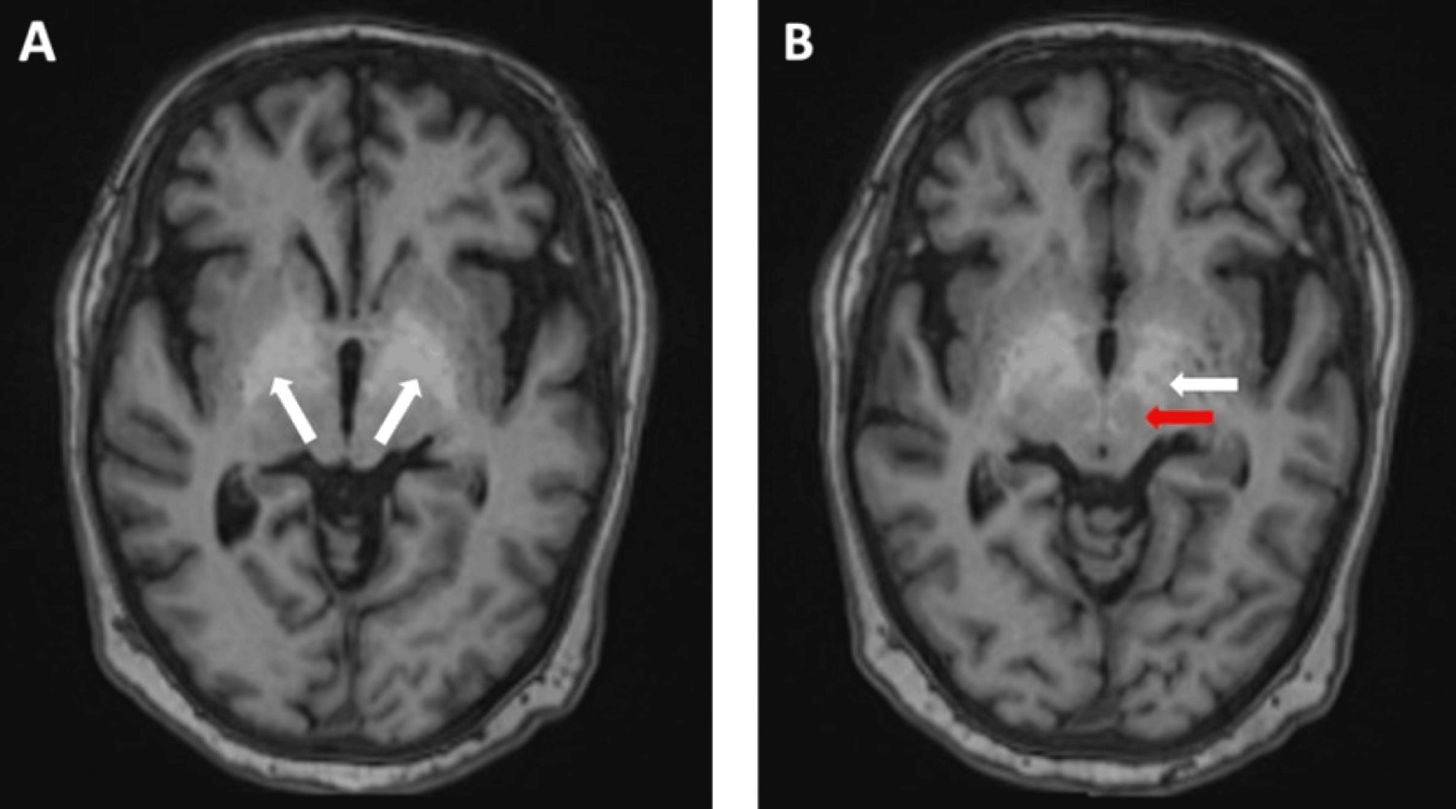
Axial T1-weighted magnetic resonance imaging (MRI) head demonstrating T1 hyperintensity in the globi pallidi (white arrows) in A and the red nucleus (red arrow) and subthalamic nucleus (white arrow) in B, characteristic of manganese (Mn) brain accumulation.

Her liver screening blood tests for genetic (haemochromatosis, Wilson disease, alpha1 anti-trypsin deficiency), autoimmune (autoimmune hepatitis and primary biliary cirrhosis, diabetes mellitus), and viral infective causes (hepatitis A, B, C, E, Epstein-Barr virus, and Cytomegalovirus) all came back negative. As per the neurologist, a magnetic resonance imaging (MRI) scan of the brain was requested which demonstrated numerous focal, and some confluent, periventricular white matter lesions within the cerebral hemispheres representing small vessel disease on the background of some mild involutional change. Of particular importance, T1 hyperintensities were seen bilaterally in the globus pallidus, subthalamic nucleus, red nucleus, and substantia nigra, which is indicative of the possibility of brain Mn accumulation (Figure 2).

**Figure 3:**
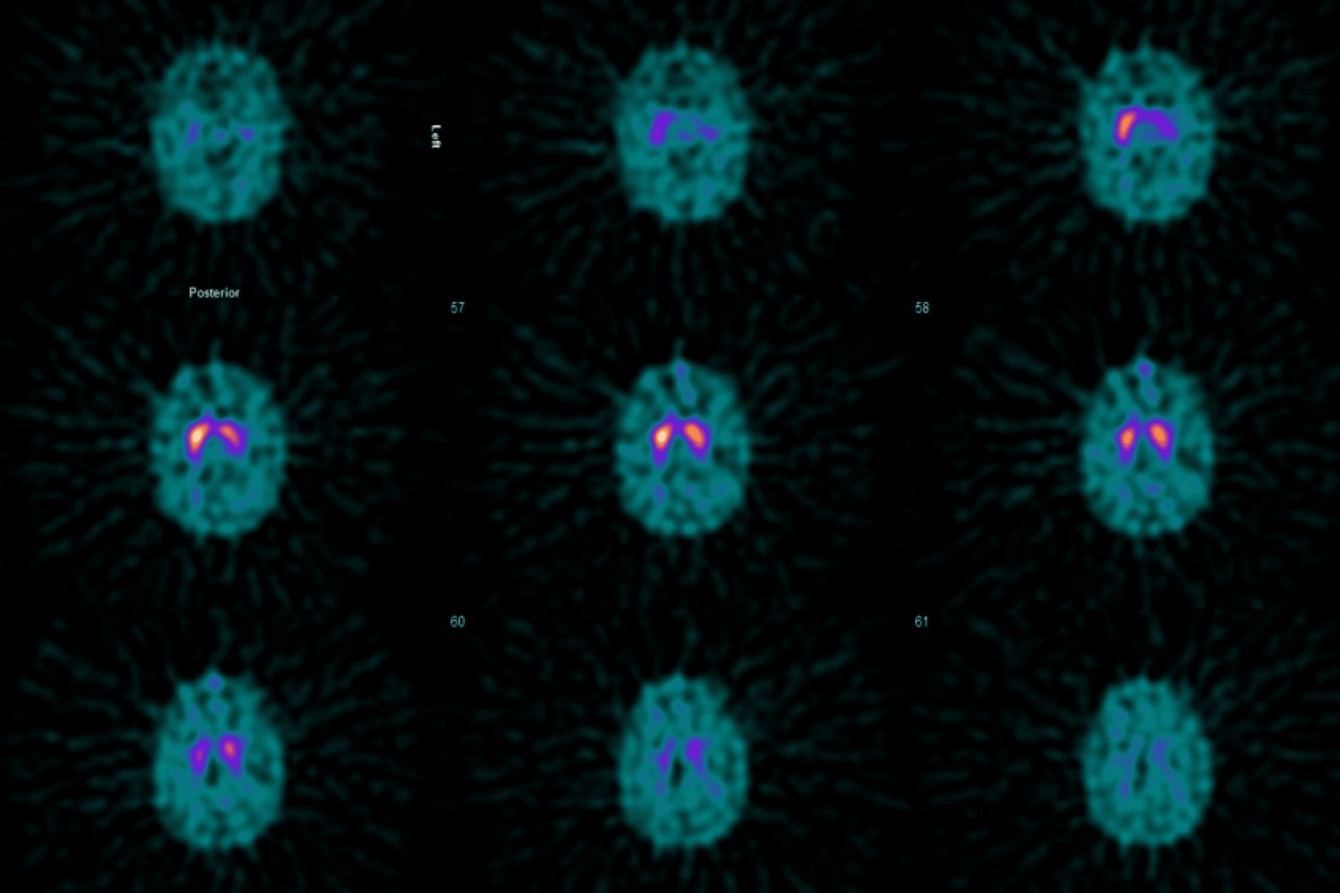
Dopamine activator transport (DAT) brain scan demonstrating normal symmetrical uptake of the tracer throughout the striata

The blood level of Mn was 726 nanomoles per litre (nmol/L) and a repeat level was 693 nmol/L with a normal range between 70 and 280 nmol/L. Additionally, normal striatal tracer uptake on the dopamine activator transport (DAT) scan indicated that her parkinsonism was not degenerative in nature due to idiopathic Parkinson’s disease or its mimics. To conclusively exclude Parkinson’s disease, a DAT scan was obtained and this demonstrated normal tracer uptake throughout the striata (Figure 3). 

A diagnosis of Parkinsonism, secondary to brain Mn deposition, with or without neuroleptic-induced Parkinsonism was made. The clinical history and presentation suggested possible environmental exposure to toxic Mn levels, exacerbated by known liver cirrhosis, as the culprit.

In consultation with the neurologist and psychiatrist, chelation therapy with ethylenediaminetetraacetic acid (EDTA) was not pursued due to the significant risk of toxicity, and whilst treatment with Levodopa was considered, the patient’s past medical history of reactive psychosis precluded this. For these reasons, the patient was maintained on lurasidone and vortioxetine.

In consultation with the hepatologist, the merits of a liver transplant were considered; however, the associated risks due to patient frailty and comorbidities were felt disproportionate, and the patient’s MELD score of 6 (range 6-40) did not meet standard liver transplant criteria. Furthermore, this intervention was unlikely to alter already established neurological deficits. For the hepatic encephalopathy, which appeared to be mild (West Haven Grading score of 2/4) lactulose 15ml three times a day was started, and Rifaximin 550mg twice a day (BD) long term was prescribed. The elderly medicine consultant also agreed with the best supportive care for the frail patient with multiple comorbidities.

The patient was discharged with a care package and arrangement for a Zimmer frame to aid in her mobility. The general practitioner was advised to periodically do blood tests, including iron studies, and arrange for oral or parenteral iron replacements as needed. 

## Discussion

MRI remains the gold standard imaging modality to diagnose neurological features of Mn toxicity with characteristic findings in T1-weighted (one tissue is bright: fat) images showing bilateral symmetric globus pallidus hyperintensities. The hyperintense T1 signals may also be seen in the cerebellar dentate nuclei, subthalamic nucleus, substantia nigra, and the tectum. On T2-weighted (two tissues are bright: fat and water) MRI, the findings are diffuse white matter increased signals with the involvement of the corticospinal tracts and subcortical focal hyperintense T2 lesions [14]. The characteristic findings on MRI have been reported in patients with cirrhosis of the liver where the serum concentration of Mn is up to three times higher than that in healthy individuals, associated with inadequate hepatic elimination [14,15]. Similar findings have been reported in patients with long-term parenteral nutrition, Crohn’s disease, and long-term use of proton pump inhibitors [14]. Liver transplantation has been reported to be associated with restoration of the liver function by blood tests and improvement in MRI T1- and T2-weighted images to even normalisation of image findings [15]. 

Today most concepts on mechanism, pharmacokinetics, pharmacodynamics, and associations of manganese with iron are established by the invitro laboratory studies. Mn has been reported in laboratory studies to promote endoplasmic reticulum stress leading to cytotoxicity in nigral dopaminergic neurons. Mn is reported to do so by using cellular surrogates of human disease in the established mouse neural precursor line namely SN4741 cells in laboratory settings [16] and neuroblastoma [17]. Interestingly, Thompson et al. established a mechanistic role for divalent metal transporter-1 (DMT1) in manganese transport by using a rat model in experimental studies and showed that levels of DMT1 increase with iron deficiency [18]. Kim et al. showed increased Mn absorption across the olfactory tract to the brain suggesting uptake of Mn in brain tissues responding to low iron levels [19,20]. This interaction between iron deficiency and enhanced brain Mn levels and brain accumulation is critically essential as Iron deficiency is the most prevalent nutritional deficiency worldwide [20].

In this patient, the comorbidity of CLD due to MASLD along with repeated exposure and iron deficiency remains the main reason for chronic Mn toxicity due to possible impaired elimination of Mn via the hepatic route.

Her good liver synthetic function reflected by a low MELD score of 6 along with her multiple comorbidities suggested liver transplantation was not in her best interest. Her concurrent IDA may have led to Mn neurotoxicity. Early recognition of these findings especially in the presence of iron deficiency may warrant evaluation for Mn toxicity in patients with CLD. 

## Conclusions

This case highlights the effects of Mn deposition on neurotoxicity in patients with liver cirrhosis. It also highlights that patients with liver cirrhosis with neurological symptoms may have causes other than hepatic encephalopathy contributing to their symptoms, including IDA. A detailed history with a high index of suspicion followed by a full neurological assessment may guide investigations. This may be particularly important in frail patients with multiple comorbidities.
